# Free and Forced Rossby Waves in the Western South China Sea Inferred from Jason-1 Satellite Altimetry Data

**DOI:** 10.3390/s8063634

**Published:** 2008-06-01

**Authors:** Xiangyu Wu, Qiang Xie, Zhigang He, Dongxiao Wang

**Affiliations:** Key Laboratory of Tropical Marine Environmental Dynamics, South China Sea Institute of Oceanology, Chinese Academy of Sciences, Guangzhou, P.R. China; E-Mails: wuxy@scsio.ac.cn (X.W.); gordonxie@scsio.ac.cn (Q.X.); zghe@scsio.ac.cn (Z.H.)

**Keywords:** intra-seasonal signal, South China Sea, Rossby wave, sea surface height

## Abstract

Data from a subsurface mooring deployed in the western South China Sea shows clear intra-seasonal oscillations (ISO) at the period of 40∼70 days. Analysis of remotely-sensed sea surface height (SSH) anomalies in the same area indicates that these ISO signals propagate both eastward and westward. Time-longitude diagrams of ISO signals in SSH anomalies and wind-stress curl indicate that the eastward propagating SSH anomalies is forced by wind-stress curl. This is also confirmed by lag correlation between SSH anomalies and the wind-stress-curl index (wind stress curl averaged over 109.5°E -115°E and 12°N -13.5°N). Lag correlation of SSH anomaly suggests that the westward propagating signals are free Rossby waves.

## 1. Introduction

Intra-seasonal variability is the most dominant mode in the tropical atmosphere [Madden and Julian, 1972], with period of 30∼90 days. Intra-seasonal signals are, therefore, very useful for forecasting tropical climate [Matthews et al., 1996]. Previous investigations of intra-seasonal oscillations (ISO) over the South China Sea (SCS) indicate that there are two bands of period (10∼25 and 30∼60 days) with different spatial structures during boreal summer [Kajikawa and Yasunari, 2005]. ISO generally consist of alternating episodes of active and suppressed atmospheric convection; it moves northward in the eastern Indian Ocean and the SCS, where air-sea interaction may be an important component of this monsoonal ISO [Sengupta et al., 2001]. Mao and Chan [2005] suggested that the 30∼60-day and 10∼20-day intra-seasonal modes are essential in controlling the SCS summer monsoon (SM). The 30–60-day oscillations of the SCS SM exhibit a trough–ridge seesaw, with anomalous cyclones (anticyclones), along with enhanced (suppressed) convection, migrating northward from the Equator to mid-latitudes. The 10∼20-day oscillations manifest anticyclone-cyclone systems over the western tropical Pacific, propagating northwestward into the SCS. The flow patterns in connection with a non-active period to an active period resemble those associated with a short Rossby wave.

**Figure 1.**
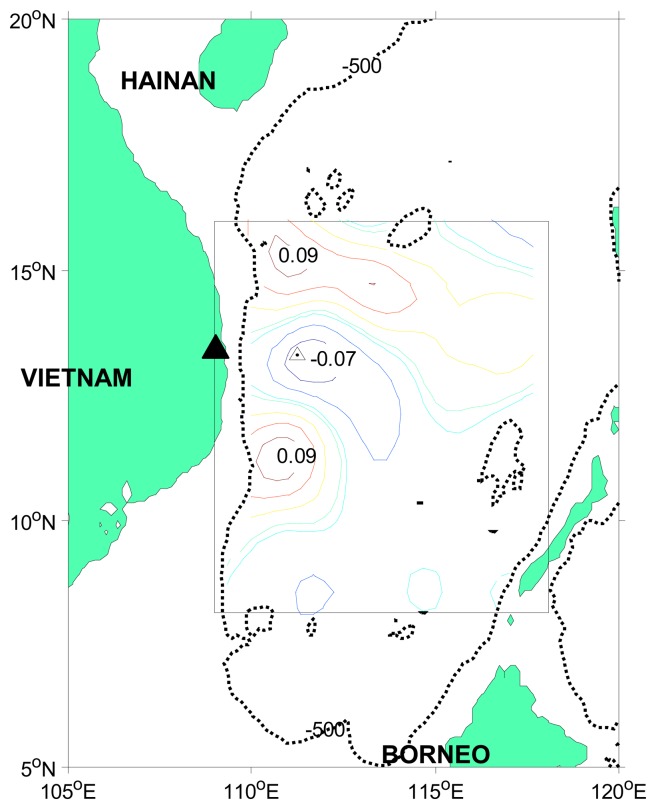
Bathymetry of the South China Sea. The thin black line indicates the 500-m isobath, the rectangular box shows the study area, the contours inside the box show one of the intra-seasonal EOF spatial patterns. Triangle marks the location of the mooring buoy.

The SCS is a large marginal sea in the Southeast Asia, with a total area of 3.5 million km2 and the average depth of over 2000 m (Fig.1). The SCS is controlled by the Southeast Asia monsoon system, and the basin-scale circulation in the upper ocean is regulated by the monsoon [Wyrtki, 1961]. Sea surface temperature (SST) and subsurface temperature in the SCS have intra-seasonal variations [Zhou and Gao, 2002], with amplitudes from 0.3°C to 0.5°C [Kawamura et al., 1988; Krishnamurti et al., 1988; Zhou et al., 1995]. The atmosphere has a memory shorter than the ocean. At the same time, SST, ocean currents and SSH anomalies can affect the atmospheric circulation, most likely through Rossby-wave adjustments [Xie et al., 2007]. In some regional circulation studies based on TOPEX/Poseidon (T/P) altimetry data, mesoscale variations in SSH anomalies were assumed to be a response to wind forcing [Liu et al., 2001].

Recently, Isoguchi and Kawamura [2006] showed that the summer upwelling/blooms off the South Vietnam coast and their offshore spreading occurs in sync with the intra-seasonal cycle of SST. Based on a global daily altimetry dataset Chu and Fang [2003] found that the westward Rossby waves weakened through the pathway from the western Pacific into the northern SCS (between 16°N-20°N). The phase speed of these waves was estimated as 5∼8 cm/s. Yang and Liu [2003] also studied forced Rossby waves in the northern SCS. Their results indicate that SSH anomalies can be interpreted in terms of the forced Rossby waves with an annual period. These waves originate off the northwest Philippine Islands and propagate northwestward to Guangdong coast with a speed of approximately 5 cm/s. Most importantly, their study revealed that the wind-stress curl dominates the occurrence and propagation of the forced Rossby waves.

Our study is focused on the western SCS from 8°N to 16°N and 109°E to 118°E. In this area, the East Vietnam eddy [Qu, 2000; Liu et al., 2001] is driven by monsoon. A southward along the western boundary current occurs in winter [Liu et al., 2004]. In summer season a wind jet drives a dipole-like eddy pair [Xie et al., 2003], i.e., a cyclone gyre in the north and an anti-cyclone gyre in the south [Gan et al., 2005].

In this study, we analyze wind and SSH anomalies over the intra-seasonal time scale. Data and methods are described in Section 2. In Section 3, SSH anomalies are analyzed using EOF method, and wind-stress curl (90∼day high-pass) data is also analyzed. A summary and some discussions are presented in Section 4.

## 2. Data and method

A subsurface mooring was deployed at 110°31.246′E and 13°59.112′N (Fig. 1), with data collected from May 2004 to September 2005. The depth of the upward-looking Acoustic Doppler Current Profiler (ADCP) is 500 m, with the exact measurement range of 48-500 m and bin length of 8 m. In this study, we averaged mooring data (varies in time) over the depths of 48-80 m (hereafter mixed-layer-averaged velocity). The climatological mixed-layer-depth inferred from hydrographic profiles is 80 m and the ADCP data in the upper 40 m is unreliable. A 48-hour low-pass filter was used to remove the tide signal in the ADCP data [Wu et al., 2005].

Jason-1 is the first follow-on to the highly successful TOPEX/Poseidon mission that measured ocean surface topography to an accuracy of 4.2 cm, enabled scientists to forecast the El Niño events, and improved understanding of ocean circulation and its effect of global climate. Altimeter observations from the Jason-1 weekly mission on 1/3°×1/3° grid were used to extract intra-seasonal variations of SSH anomalies from May 2004 to Sep 2005 using EOF method. Sea surface winds used are from the QuikSCAT. QuikSCAT 3-day-averaged daily data is available on a 0.25°×0.25° grid. The high resolutions in both space and time of the satellites data are adequate for the investigation of the intra-seasonal variability in the western SCS.

**Figure 2.**
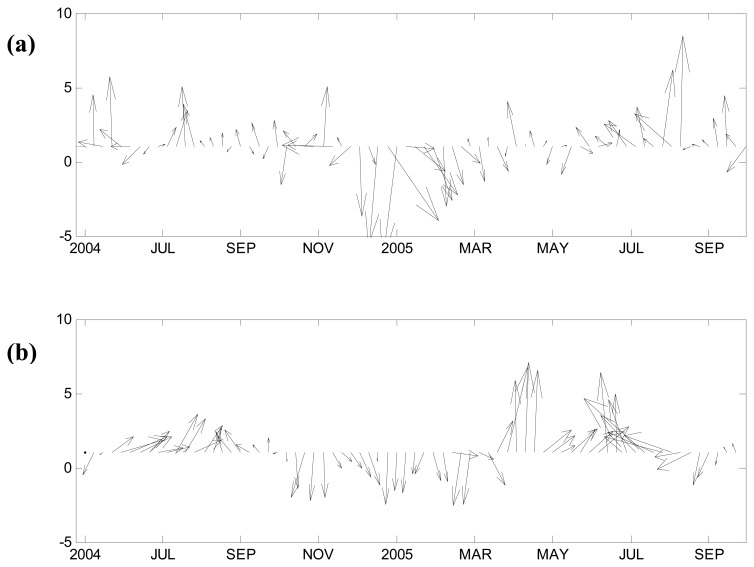
Stick plots of the mixed-layer velocity, in units of cm/s. **(a)** Geostrophic velocity calculated from T/P data. **(b)** Mooring-recorded velocity (average over 48-80 m).

The altimetry-derived geostrophic currents and mixed-layer-averaged velocities compared well (Fig. 2), both showing the variability in the upper ocean of the study area. This gives us confidence to use T/P and ADCP data for further analysis.

## 3. Results and Discussion

### 3.1 Identifying intra-seasonal oscillation

The mooring buoy was located north of the Vietnam cold eddy (Fig. 1), and provided useful information about the western SCS. The power spectra of velocity show that 40∼70-day signals are present in both the zonal and meridional components (not shown).

EOF analysis of SSH anomalies in the study area indicates that the five leading modes can explain 72.4% of the total variance (Table 1), so the main features of the SSH anomalies can be described by the first five EOF modes. The period of EOF1 is about 120 days, while EOF2 to EOF5 are intra-seasonal modes, whose cumulative variance is 42.5% of the total variance (Table 1).

**Figure 3.**
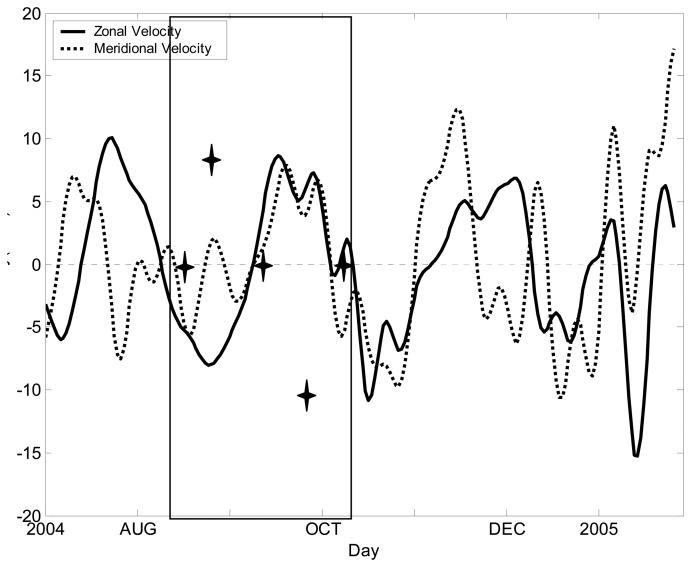
Time series of 10∼90-day band-passed mixed-layer velocity (averaged over 48-80 m) measured by the mooring. Two vertical lines indicate a complete cycle for a case study. The asterisks stand for five typical stages of the case study, with the snapshots of the SSH anomaly shown in Fig.4.

We applied 10∼90-day band-pass to the mixed-layer-averaged velocity, which contains intra-seasonal oscillations, and then chose a complete cycle of averaged U- and V-fields (enclosed by the two vertical lines in Fig. 3). In order to identify the signals of intra-seasonal SSH anomalies, we used EOF2 through EOF5 from this complete cycle as a case study.

### 3.2 Case study

**Figure 4.**
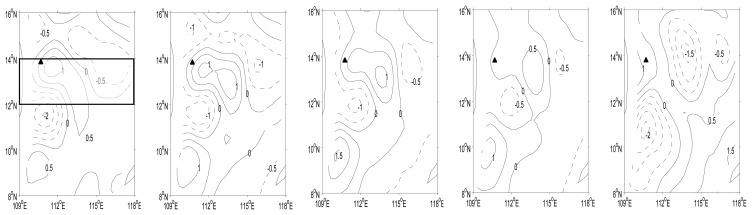
Snap-shots of intra-seasonal EOF patterns (composition of EOF phase 2 to 5) of SSH anomaly. The triangle indicates the mooring position. The sampling time was chosen based on mooring data time series in Fig. 3. Two horizontal lines will be used for meridian average in the longitude-time plot.

The composition of intra-seasonal EOF spatial pattern is shown in Fig.4. Three SSH anomaly centers in the west are obviously eastward. The two positive areas between 12°N and 14°N and between 8°N and 10°N became stronger as they propagated eastward. They reached their peak values in phase 3, 1 cm in the north and 1.5 cm in the south. The negative area in the middle had weak eastward propagation and weakened in time; it also reached its peak value of -1 cm in phase 3. In the northeast, the cyclone showed clear westward propagation starting from phase 3, and it reached its peak value of -1.5 cm in phase 5. During phase 5, the study area is controlled by the northeast monsoon; the SSH anomalies signals and circulations adjusted to the wind field. The above results clearly show that both eastward and westward propagations took place in our study area.

**Figure 5.**
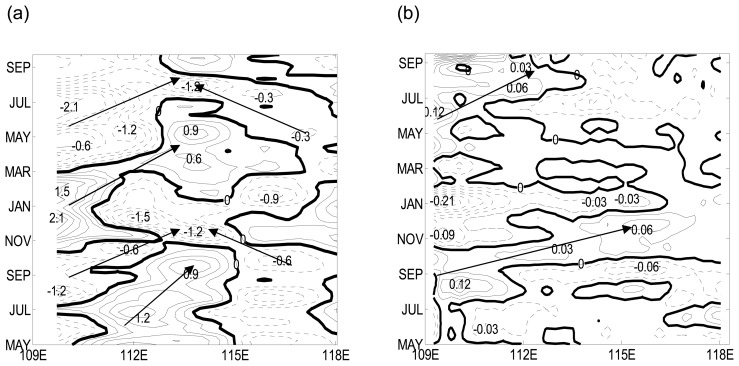
Longitude-time plots for May 2004 to Sep 2005. **(a)** 10∼90-day band-passed QuikScat wind-stress curl averaged over 12°N-13.5°N (units in N *m*^-1^). **(b)** Intra-seasonal anomalies of TOPEX/Poseidon SSH signals averaged over 11.67°N-13.67°N (units in m).

According to the case study above, the intra-seasonal SSH anomalies in the study area can propagate eastward or westward. Westward propagation of planetary waves has been clearly identified in the open ocean [e.g., Jacobs et al., 1994; Chelton and Schlax, 1996]. It is well known that there are free Rossby waves in SCS. The westward signals may represent free Rossby waves, while the eastward signals might be due to the ocean-to-atmosphere feedback. We carried out time-longitude analysis in the study area. For each section the signals are averaged over a small latitudinal band, as indicated in Fig. 4, Fig. 5a shows four eastward propagating packages and two westward packages with abrupt phase reversals identified from SSH anomalies for latitudinal band between 11.67°N-13.67°N. In 2004, a negative peak started near 110°E in August and propagated to 114°E in about five months; however, at the same time, westward propagation with a negative peak appeared between 115°E-118°E. These two propagating waves converged around 114°E, and the westward propagating negative SSH anomaly was enhanced to -1.2∼ -1.5 cm (Note that it is in term of zonal group velocities of Rossby waves).

Two months before the negative signal appeared, a positive peak around 112°E moved eastward, and it met with a westward negative signal at 115°E. As a result, the positive signal is completely wiped out. In 2005, a negative signal of -0.6 cm started in April (nearly four months earlier than the corresponding negative signal in 2004). In August, it propagated to around 114°E. At the same time, a westward signal of -0.3 cm appeared between 117°E-118°E.

Ahead of this negative signal, there was a positive signal, which appeared around 109°E in December 2004. At 112°E, it met with the westward negative signal. As a result, this positive SSH anomaly vanished. The intensity and time of appearing of the SSH anomalous signals in 2004 and 2005 were different, and such differences may be due to the effect of wind forcing in this typical wind field region of SCS.

To find out the cause of such difference we carried out time-longitude analysis of 10∼90-day bandpass-filtered wind-stress-curl signals in the same area as the SSH anomalies mentioned above. As shown in Fig. 5b, four sets of eastward propagating signals in wind-stress curl can be identified between May 2004 and in May 2005 between 109°E and 118°E. In 2004, a negative package around 110°E moved eastward in May and a positive package of 0.12m appeared in July, and it moved to 118°E in November and the peak value became 0.06m. In November 2004 a negative package around 109°E and moved eastward. In 2005, the positive signal package of 0.12m appeared in May, and in September it propagated to 114°E.

To sum up, the two negative eastward propagating of SSHA signals each year seems have closely correspond to the eastward propagating signals in wind-stress curl discussed above. But the other two positive eastward propagating of SSHA signals seems determined by more elements besides eastward propagating of wind stress curl signals.

South of 18°N in SCS, the SSHA obtained from T/P altimetry and steric height calculated from Levitus monthly climatology exhibit eastward zonal migration, which appear to be forced by eastward migration of wind stress curl [Liu et al., 2001]. Surface wind is a likely mechanism generating SSH anomalies. Mesoscale eddies can be generated by the combination of wind stress curl anomaly and baroclinic instability. There is another explanation in terms of the horizontal advection due to the background large-scale circulation, thus both baroclinic instability and mean flow advection will be discussed in our other studies.

### 3.4 Hypothesis

The lack of obvious westward propagating wind-stress-curl signals suggests that the westward propagating package of SSH anomalies in Fig.5a is a free Rossby wave. A positive wind-stress-curl anomaly in summer generates Ekman upwelling and cooling in the upper ocean, generating a negative SSH anomalies; while a negative wind-stress-curl anomaly in winter has an opposite effect. The SCS monsoon, affected by the SCS circulation, belongs to the planetary-scale atmospheric circulation system; it belongs to a much larger system, the South Asian monsoon. Studies of the northeast SCS have shown that the wind-stress curls could be one of the major factors trigging the intra-seasonal variations in velocity fields [Wu et al., 2005]. The oceanic variability in the area studies in this report is a forced response in forms of the eastward Rossby waves.

**Figure 6.**
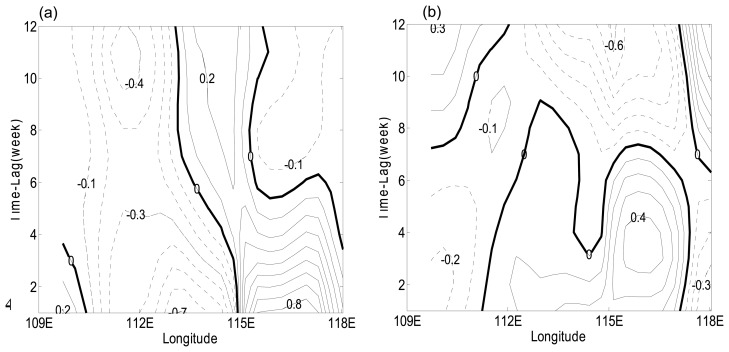
Lag correlations for the period from May 2004 to Sep 2005. The time lag is one week. **(a)** Lag correlations between SSH anomalies and the eastern SSH anomaly index (SSH anomalies averaged over 115°E -119°E and 11.67°N-13.67°N). **(b)** Lag correlations between SSH anomalies and the wind-stress-curl index (wind-stress curl averaged over 109.5°E -115°E and 12°N -13.5°N).

To demonstrate the mechanism responsible for the generation of these waves we calculated the following indexes. The eastern SSH anomalies index is defined by averaging SSH anomalies over 115°E-119°E and 11.67°N-13.67°N, while the wind-stress curl index is defined by averaging wind-stress curl anomaly over 109.5°E-115°E and 12°N-13.5°N. The data used for calculating lag correlation is one and a half year long, good for a maximum of 12-month lag correlation. Lag correlation between SSH anomalies and eastern SSH anomaly index (Fig. 6a) suggests that the westward propagating package is a free Rossby wave; while lag correlation between SSH anomalies and wind-stress index (Fig. 6b) indicates that the eastward propagating packages are forced Rossby waves forced by wind curl anomaly. According to Fig. 6a, east of 113°E the correlation between SSH anomalies and the eastern SSH anomaly index is positive and the phase speed is estimated as 4.8 cm/s, which suggests a free Rossby wave. According to Fig. 6b, the SSH anomalies and wind-stress-curl index have negative correlation from west to east, which suggests that ISO in the SSH anomalies are driven by intraseasonal variability in the wind-stress curl, and the phase speed is estimated as 4.6 cm/s.

Based on analysis of remote-sensing and mooring data, we conclude that intra-seasonal SSH anomalies in the southern SCS can be interpreted as forced Rossby waves or free Rossby waves. The eastward intra-seasonal signals are forced by intra-seasonal variations of wind-stress curl, while the westward intra-seasonal signals represent planetary waves. The meridional and zonal currents have clear intra-seasonal variations (Fig. 3), whose complete life cycle shows that the intra-seasonal signals can propagate either eastward or westward. These propagations may affect the circulation in southern SCS. The complete cycle of the variation can be examined through a carefully selected case study (Fig.4). There are two opposite propagations within a complete cycle, which is an ocean reaction to the atmosphere in terms of Rossby waves. The consistency between wind-stress curl and SSH anomalies signals suggest that the curl be one of the major factors to trigger the intra-seasonal variations. Further lag correlation analyses suggest that the westward propagation is a free Rossby wave and the eastward propagating packages are forced Rossby waves.

## Figures and Tables

**Table 1.** Percentage of the explained variance of the five leading EOF modes.

EOF modes	Percentage of the total variance	Accumulative percentage
1	29.9%	29.9%
2	16.7%	46.6%
3	12.1%	58.7%
4	8.4%	67.1%
5	5.3%	72.4%
